# Supplementary material for: Domain-dependent strain and stacking in two-dimensional van der Waals ferroelectrics
Source: Nat Commun. 2023 Nov 7;14:7168. doi: 10.1038/s41467-023-42947-3 (PMC10630342; doi:10.1038/s41467-023-42947-3)
Supplement: Supplementary file 1 — Supplementary Information [file 41467_2023_42947_MOESM1_ESM.pdf]

Supplementary Information for

Domain-dependent Strain and Stacking in Two-dimensional van der Waals Ferroelectrics

Chuqiao Shi[1], Nannan Mao[2,3], Kena Zhang[4], Tianyi Zhang[2], Ming-Hui Chiu[2], Kenna Ashen[5], Bo Wang[6], Xiuyu Tang[5], Galio Guo[1], Shiming Lei[7], Longqing Chen[6], Ye Cao[4], Xiaofeng Qian[5,8,9], Jing Kong[2], Yimo Han[1,*]

[1] Department of Materials Science and NanoEngineering, Rice University, Houston, TX, 77005, USA

[2] Department of Electrical Engineering and Computer Science, Massachusetts Institute of Technology, Cambridge, MA 02139, USA

[3] Department of Chemical Engineering, Massachusetts Institute of Technology, Cambridge, MA 02139, USA

[4] Departments of Materials Science and Engineering, University of Texas at Arlington, Arlington, TX 76019, USA

[5] Departments of Materials Science and Engineering, Texas A&M University, College Station, TX 77843, USA

[6] Materials Research Institute and Department of Materials Science and Engineering, The Pennsylvania State University, University Park, PA, USA

[7] Department of Physics, Rice University, Houston, TX, 77005, USA

[8] Department of Electrical and Computer Engineering, Texas A&M University, College Station, Texas 77843, USA

[9] Department of Physics and Astronomy, Texas A&M University, College Station, Texas 77843, USA

[*] To whom correspondence should be addressed: Yimo Han (Email: yimo.han@rice.edu)

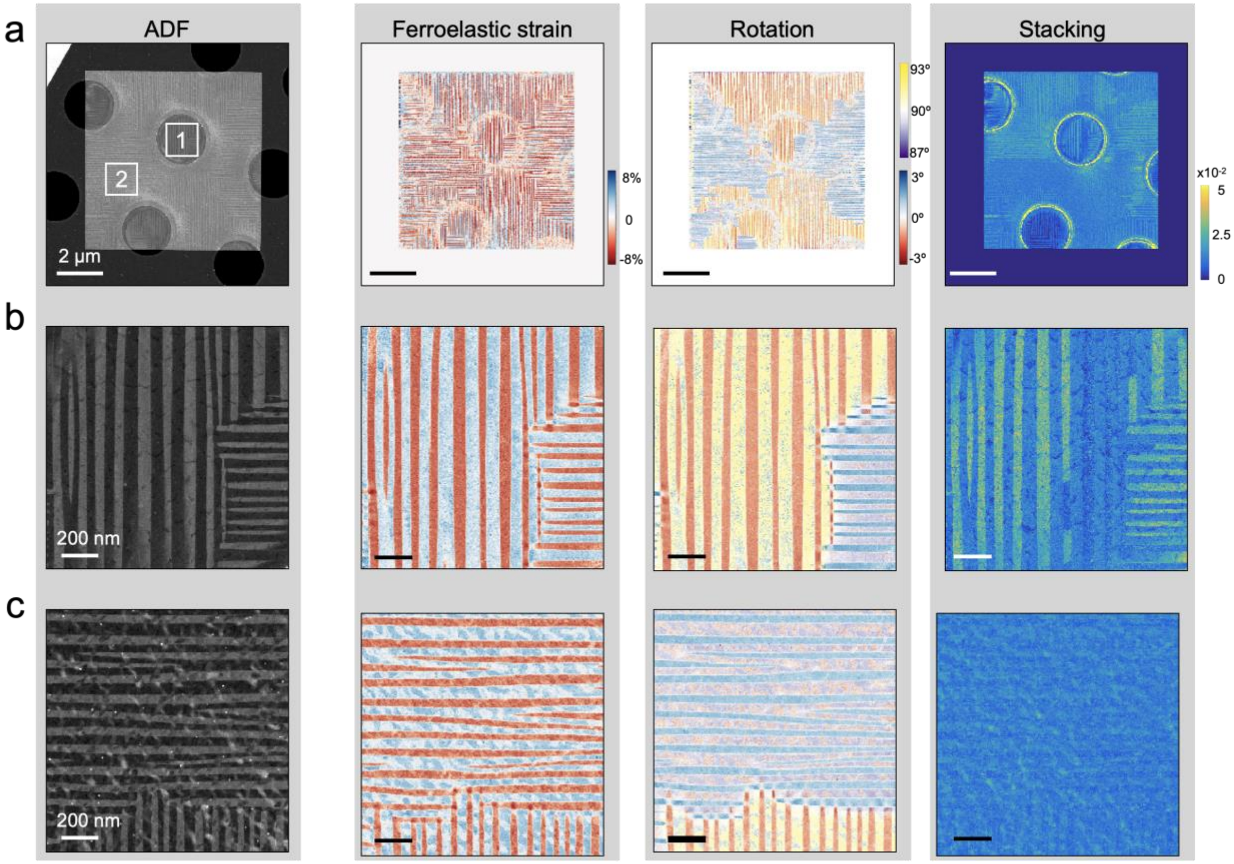

Figure S1 | 4D-STEM measurements from an additional flake (Flake I). (a) ADF-STEM images of an entire single-crystalline SnSe thin flake, with two zoomed-in regions for detail. **(b)** Corresponding ferroelastic strain maps. **(c)** Rotation maps. **(d)** (110) intensity maps indicating the stacking order.

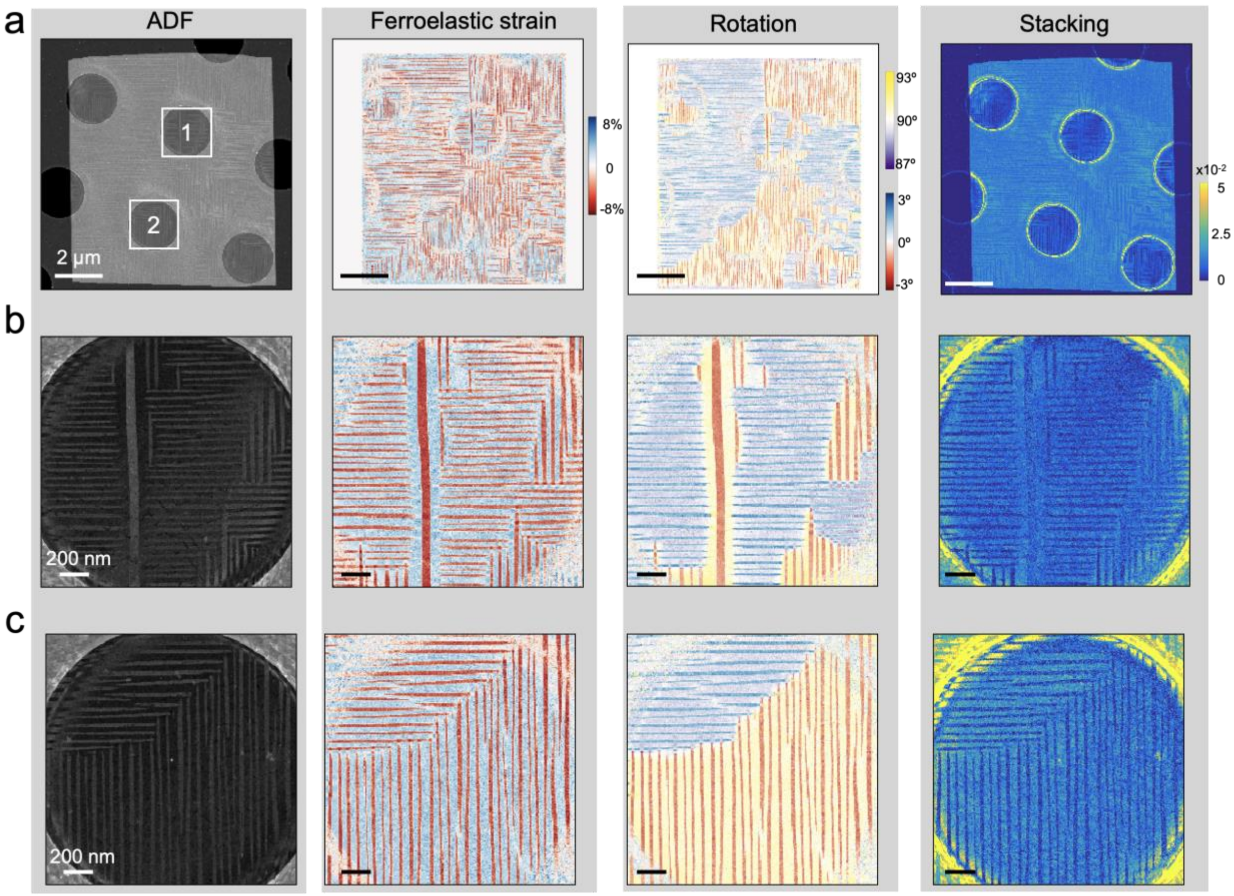

Figure S2 | 4D-STEM measurements from a different flake (Flake II). (a) ADF-STEM images of another single-crystalline SnSe thin flake, with two zoomed-in regions for detail. **(b)** Corresponding ferroelastic strain maps. **(c)** Rotation maps. **(d)** (110) intensity maps indicating the stacking order.

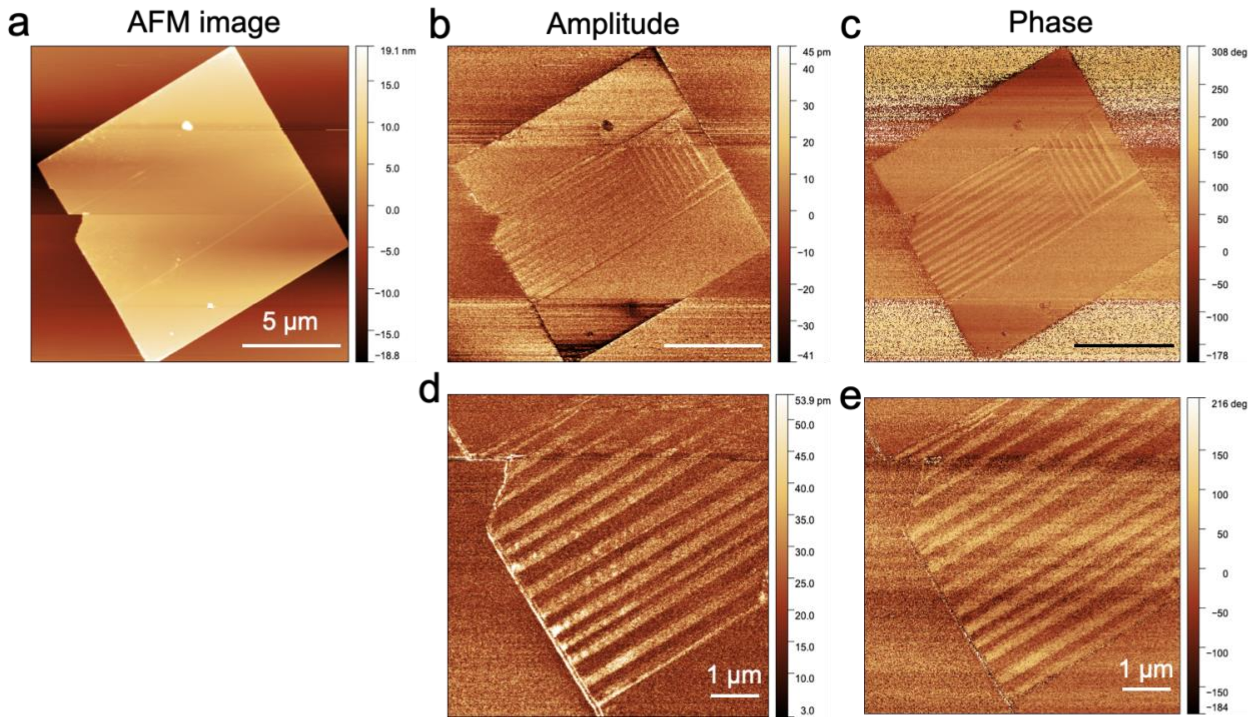

Figure S3 | Atomic force microscopy (AFM) and piezoresponse force microscopy (PFM) images of a SnSe thin flake. (a) AFM image showing the surface topography of a SnSe thin flake on its original substrate (mica surface). **(b-c)** The amplitude and phase images from PFM measurements, revealing the stripe twin domain structure within the SnSe flake, offering insights into its polarization. **(d-e)** Zoomed-in amplitude and phase images from PFM measurements which show the stripe twin domain structures.

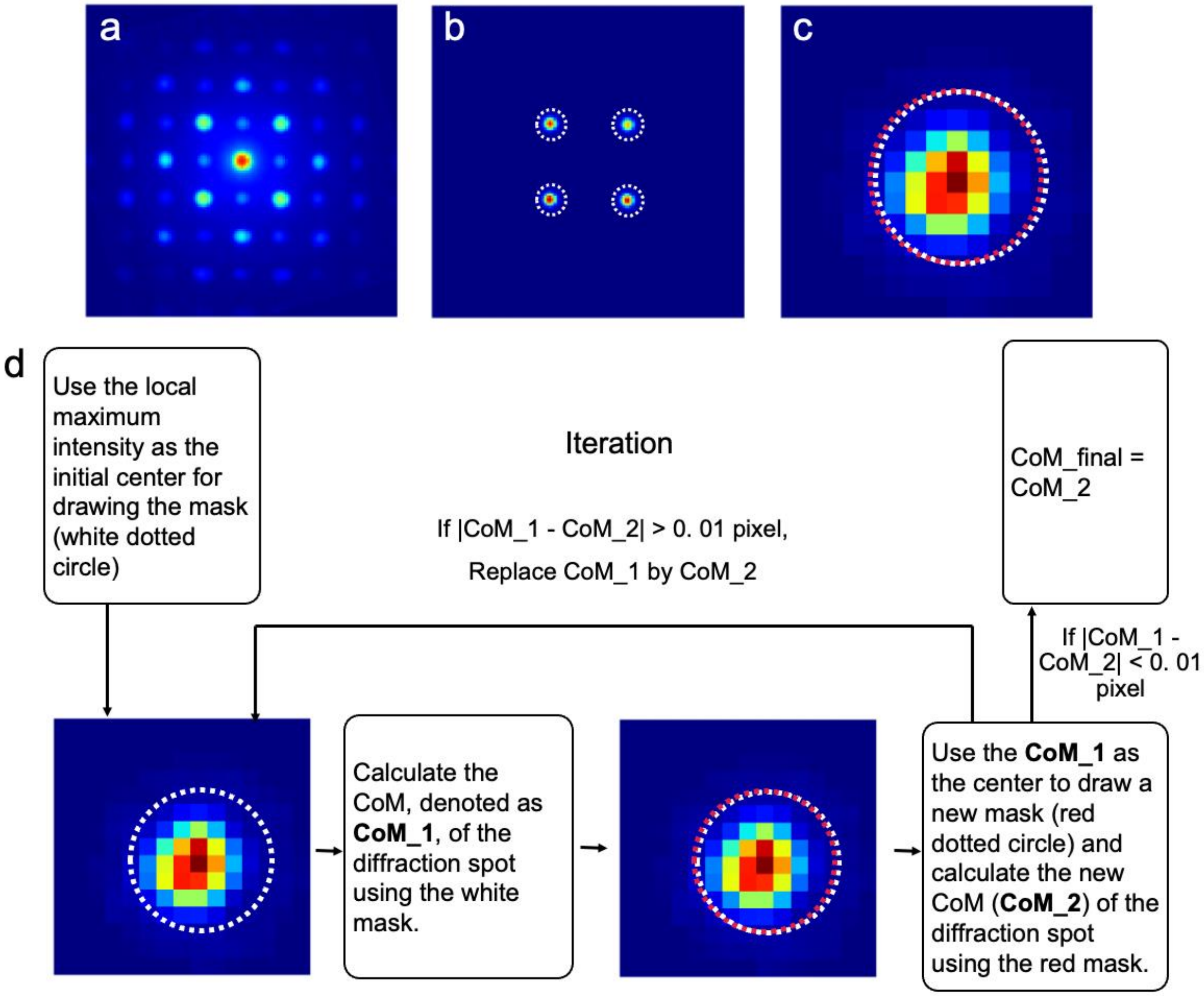

Figure S4 | Center of mass (CoM) method for 4D data processing. (a) The averaged diffraction pattern from the 4D dataset used in **Figure 1**. **(b)** Four {200} diffraction spots with their corresponding circular masks (white dotted circle). The initial center of every circular mask is set based on the pixel with the highest intensity in the mean diffraction spot. **(c)** Determination of the center for the new mask (red dotted circle) using iterative CoM calculations. **(d)** The workflow of the iterative CoM method to create adaptive masks, ensuring precise pinpointing diffraction spot positions. Upon establishing the initial mask (white dotted circle), the CoM within the mask is calculated as CoM_1. A subsequent circle mask (red dotted circle) centered on CoM_1 is applied (red). The CoM calculated using the red mask is determined as CoM_2. If the difference between CoM_2 and CoM_1 exceeds a 0.01 pixel threshold, the workflow is iterated until the difference is within the set threshold.

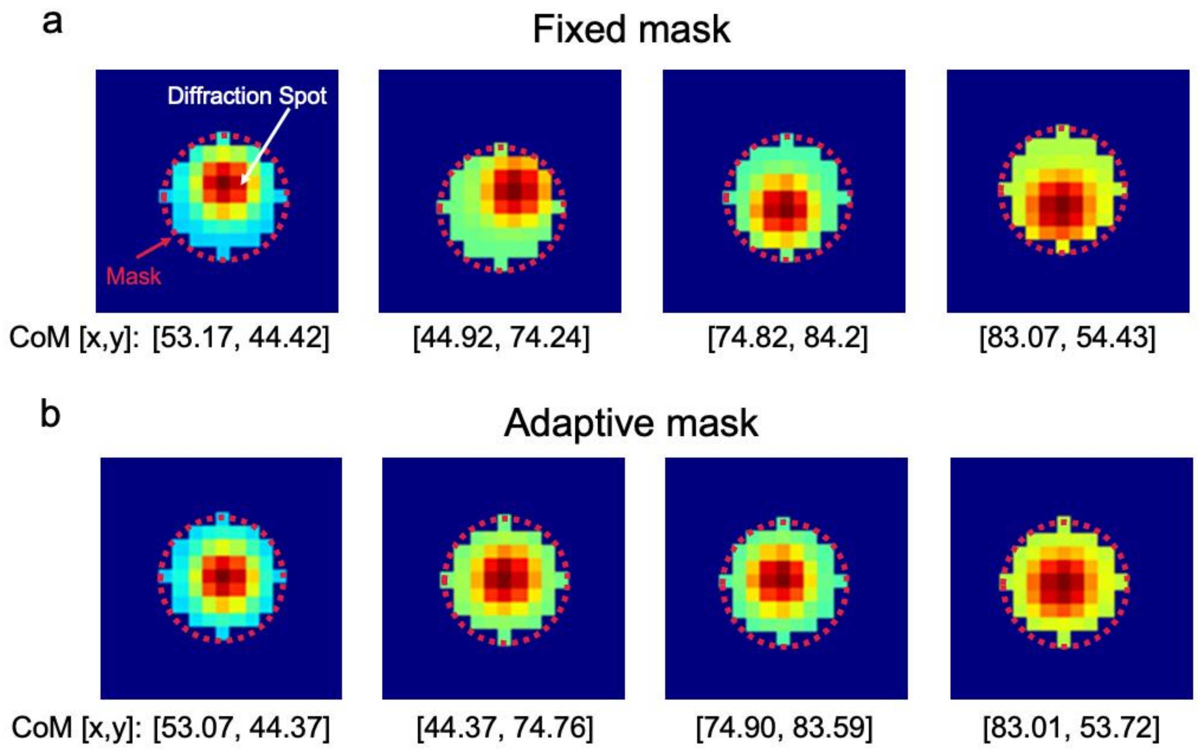

Figure S5 | Comparison of fixed-mask and the adaptive-mask methods for CoM measurements. (a) Four sample diffraction spots with the fixed masks, showing variations in different diffraction patterns. CoM values derived from these patterns are displayed below (in pixels). **(b)** Four sample diffraction spots with the adaptive masks, demonstrating evident improvements in mask alignment. A comparison of the calculated CoM values reveals that the adaptive-mask approach reduces the alignment inaccuracies caused by uneven backgrounds by up to an estimated 2%.

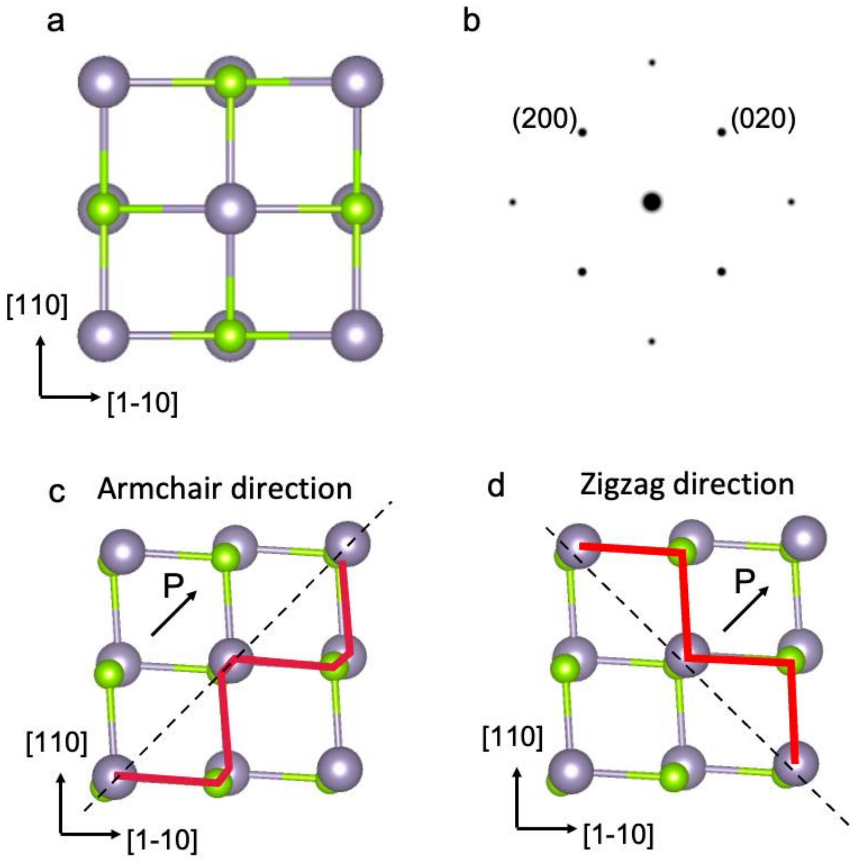

Figure S6 | Lattice schematic and simulated diffraction pattern of SnSe. (a) Lattice schematic of the paraelectric phase of SnSe, indicating no atomic displacement between Sn and Se atoms. **(b)** Simulated diffraction pattern of paraelectric phase of SnSe. **(c)** Lattice schematic of the ferroelectric (FE) phase of SnSe with the armchair direction labeled by a black dotted line. The namesake armchair shape is labeled by the red curve. **(d)** Lattice schematic of the FE phase of SnSe where the zigzag direction is labeled by the black dotted line. The red curve shows a zigzag shape.

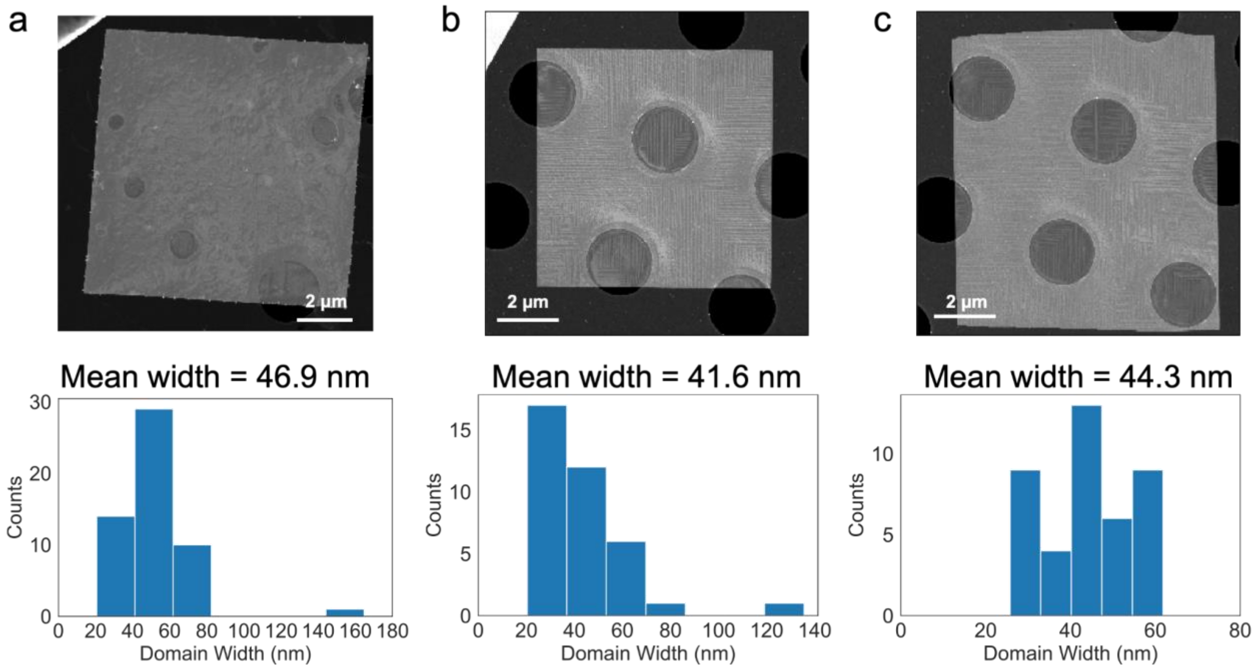

Figure S7 | Histogram showing the domain width of SnSe flakes. (a) The flake presented in **Figure 1**. **(b)** Flake I from **Figure S1**. **(c)** Flake II from **Figure S2**.

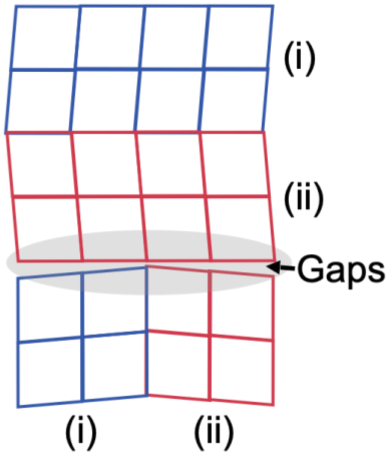

Figure S8 | Rigid lattice of superdomain boundaries (or 180° domain walls). Two 2.5° gaps appear at the boundary as a result of the 87.5° twin wall, leading to lattice mismatch.

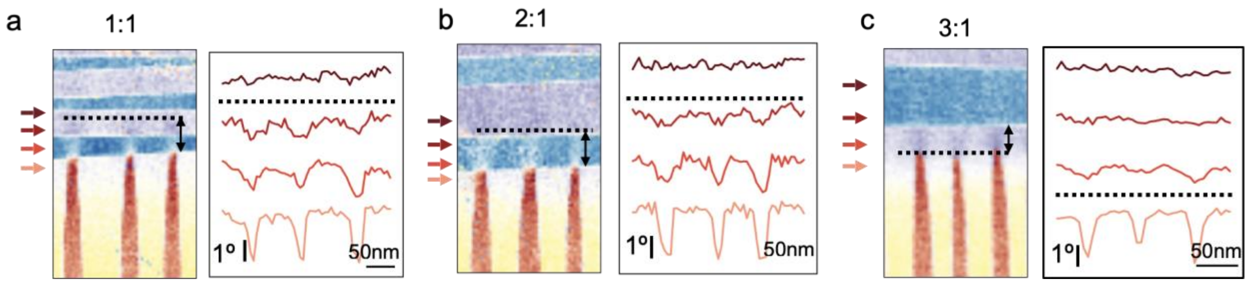

Figure S9 | Deformation length measurement. (a-b) Rotation maps of T-junctions with their intensity line profiles (in the horizontal direction) at various locations, as indicated by the red arrows to the left of the rotation maps. Four lines are displayed here as examples. The horizontal dotted lines indicate the point where the deformation becomes unobservable. The measured deformed length is marked by vertical black arrows, spanning from the intersection to the horizontal line where the deformation fades. **(c)** Rotation map and intensity line profile of superdomain boundaries with needle tips. As the needle tip structure rarely deforms the horizontal stripes, we measure the gap between needle tips (dotted line) and the intersection. We define this gas as the deformed length (indicated by vertical black arrows). Since the direction of deformation in needle tips is opposite to that in T-junctions, the deformed length in the needle tip structure is considered negative.

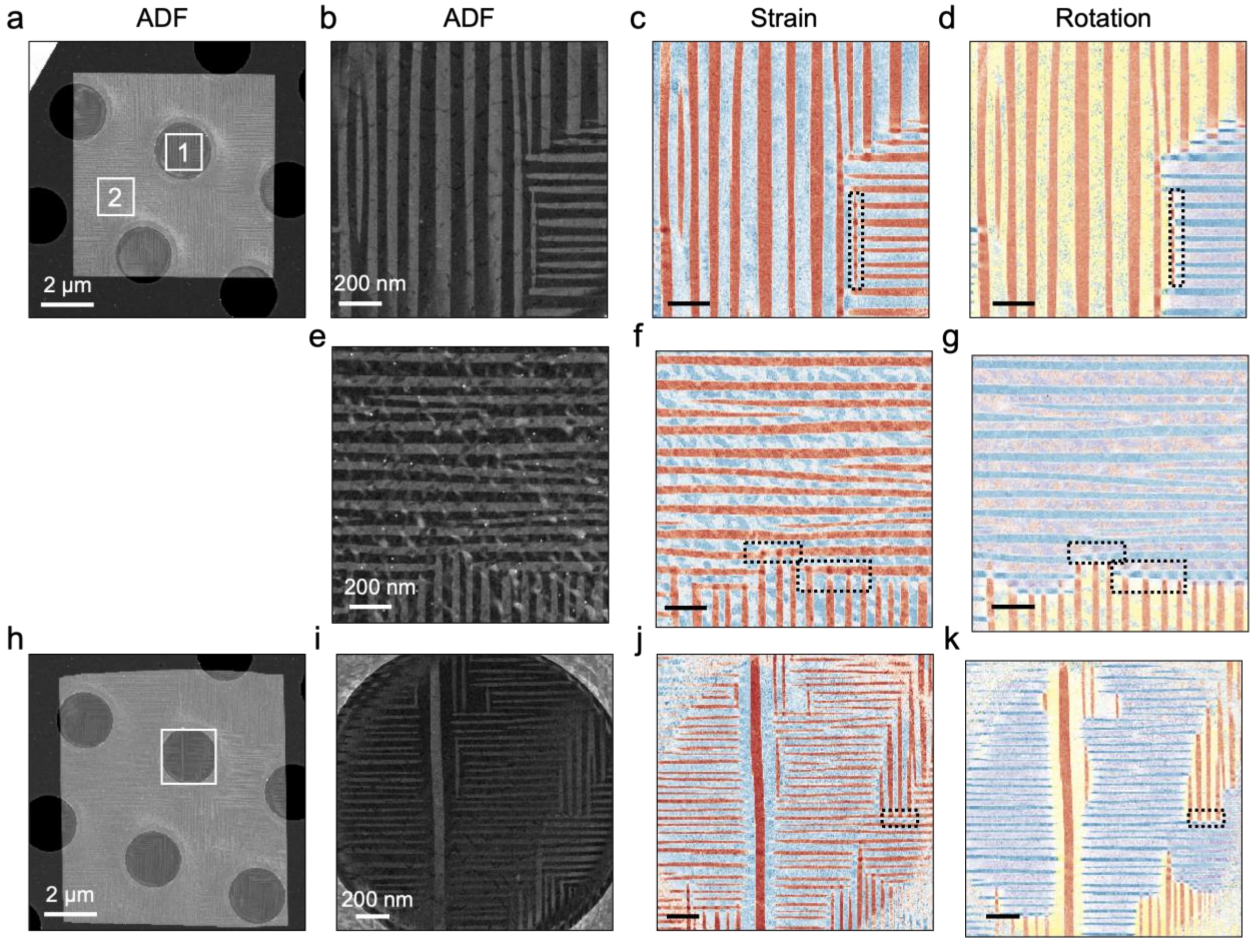

Figure S10 | T-junctions and needle tips from additional flakes. (a) Low magnification ADF image of the Flake I. **(b)** Zoomed-in ADF image of region 1 in Flake I. **(c-d)** Ferroelastic strain and rotation map of region 1. Typical examples of T-junctions are shown in the dotted box. **(e)** Zoomed-in ADF image of region 2 in Flake I. **(f-g)** Ferroelastic strain and rotation map of region 2. Additional examples of T-junctions are highlighted in the dotted box. **(h)** Low-magnification ADF image of another flake (Flake II). **(i)** Zoomed-in ADF image of a selected region in Flake II. **(j-k)** Ferroelastic strain and rotation map of the region shown in (i). In addition to examples of T-junctions (in the dotted box), we also observed numerous needle-tip structures, attributable to a relatively wide vertical stripe spanning the field of view.

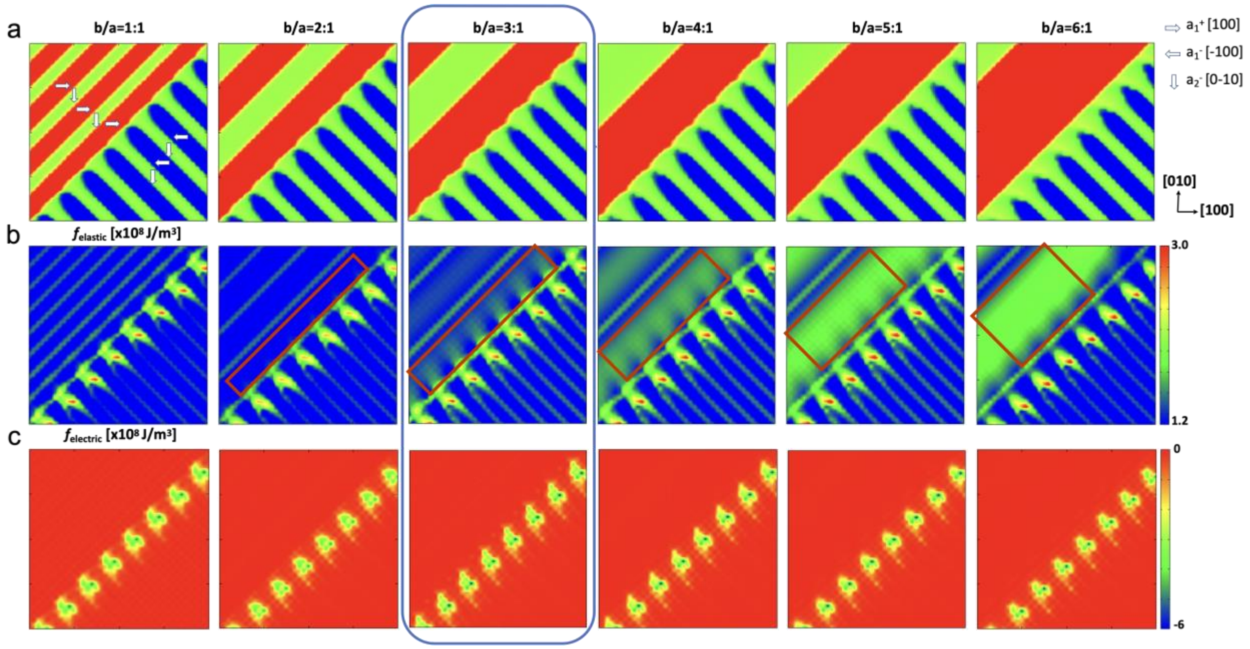

Figure S11 | Phase field simulation of superdomain boundaries with different width ratios. (a) Rotation map showing domains with varying width ratios from 1:1 to 6:1. **(b)** Simulated maps of the elastic free energy density. Notably, the elastic energy in the horizontal domains (highlighted in the red boxes) increases as the domain width ratio rises. When the elastic energy of the horizontal domain is low, it is more susceptible to deformation by vertical stripes. In contrast, higher elastic energy resists the deformation, leading to the formation of the needle tip structure. The transition zone is observed around 3:1 width ratio. **(c)** Simulated maps of the electrostatic energy density, indicating that the electrostatic energy remains unaffected by the domain width ratio.

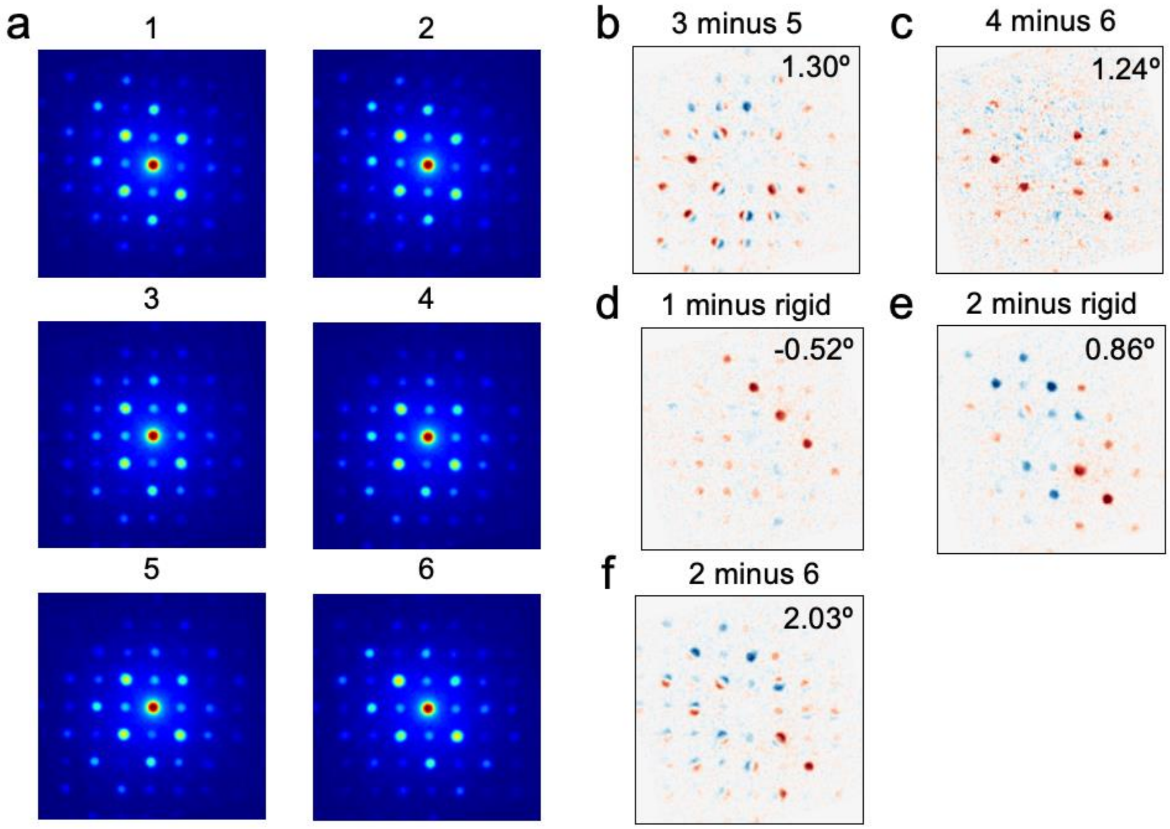

Figure S12 | Diffraction patterns at "T" junctions. (a) Diffraction patterns from the six boxed areas in **Figure 3a**. **(b-f)** Calculated differences in diffraction pattern intensity. The calculation method and the relative rotation angles are indicated above.

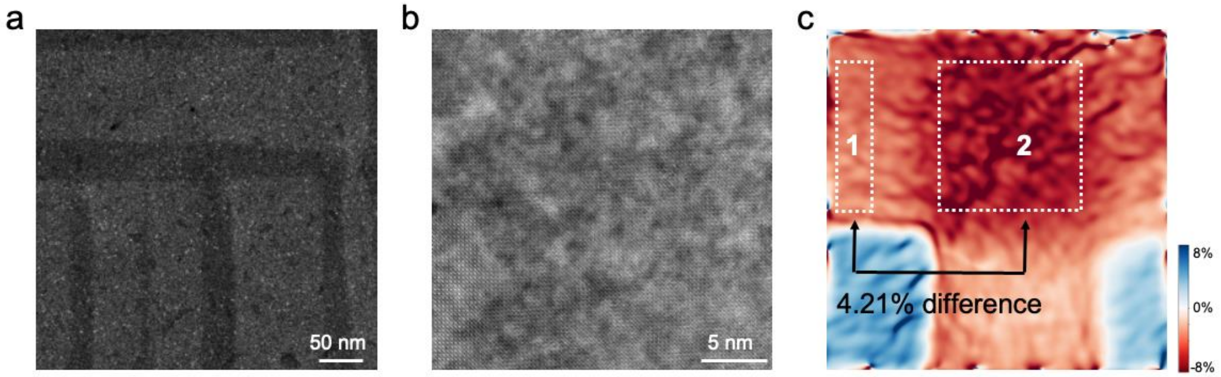

Figure S13 | Strain measurements from atomic resolution ADF-STEM images. (a) Low-magnification ADF image of T-junctions. **(b)** Atomic resolution ADF image of a T-junction, labeled in the white box in (a). **(c)** Ferroelastic strain map, calculated by geometric phase analysis (GPA) from the atomic resolution image in (b). The strain difference is 4.21% comparing the deformed T-junction and intact neighboring region. This aligns with the strain measured from 4D-STEM. The detailed calculation of strain using GPA method is shown in **Fig. S14**.

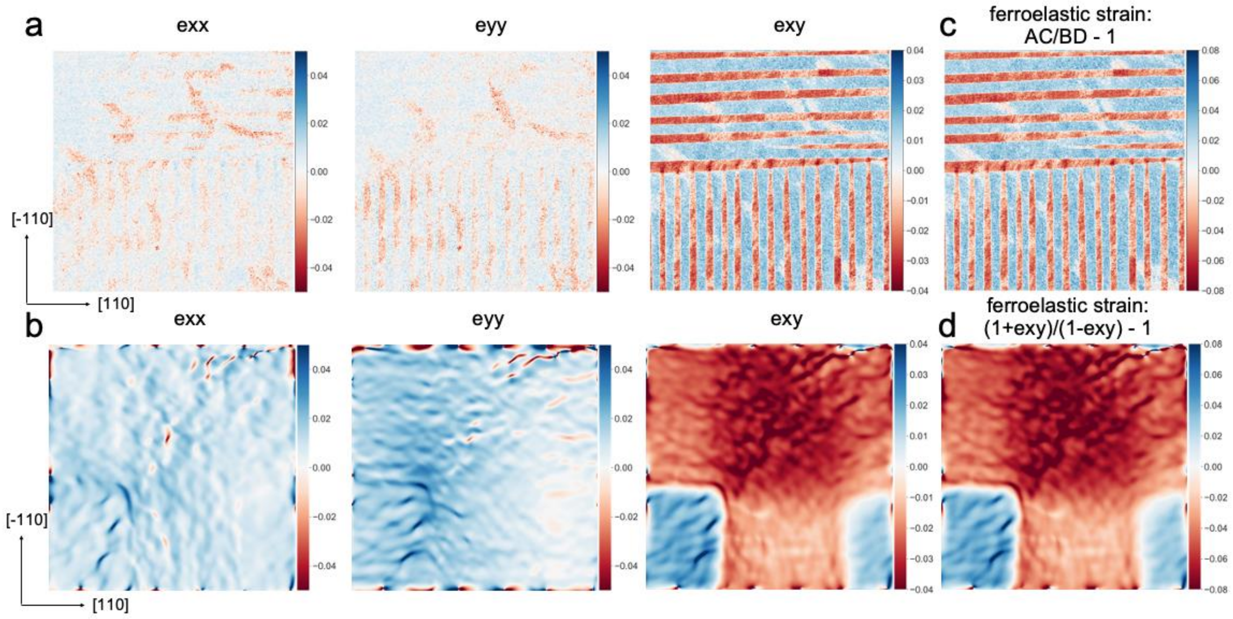

Figure S14 | Comparison between 4D-STEM and GPA strain mapping. (a) Strain maps (e_{xx}, e_{yy}, shear, and rotation) are generated from the 4D-STEM data using a conventional method[1]. [110] and [-110] vectors are chosen as the basis for strain matrix calculation. **(b)** Strain maps of a T-junction from GPA. The uniaxial and shear strain maps are consistent with the 4D-STEM results. **(c)** The ferroelastic strain map calculated using our method based on 4D-STEM. **(d)** The ferroelastic strain determined from the shear map in the GPA results, which represents the diagonal deformation (for further details, refer to **Fig. S28**).

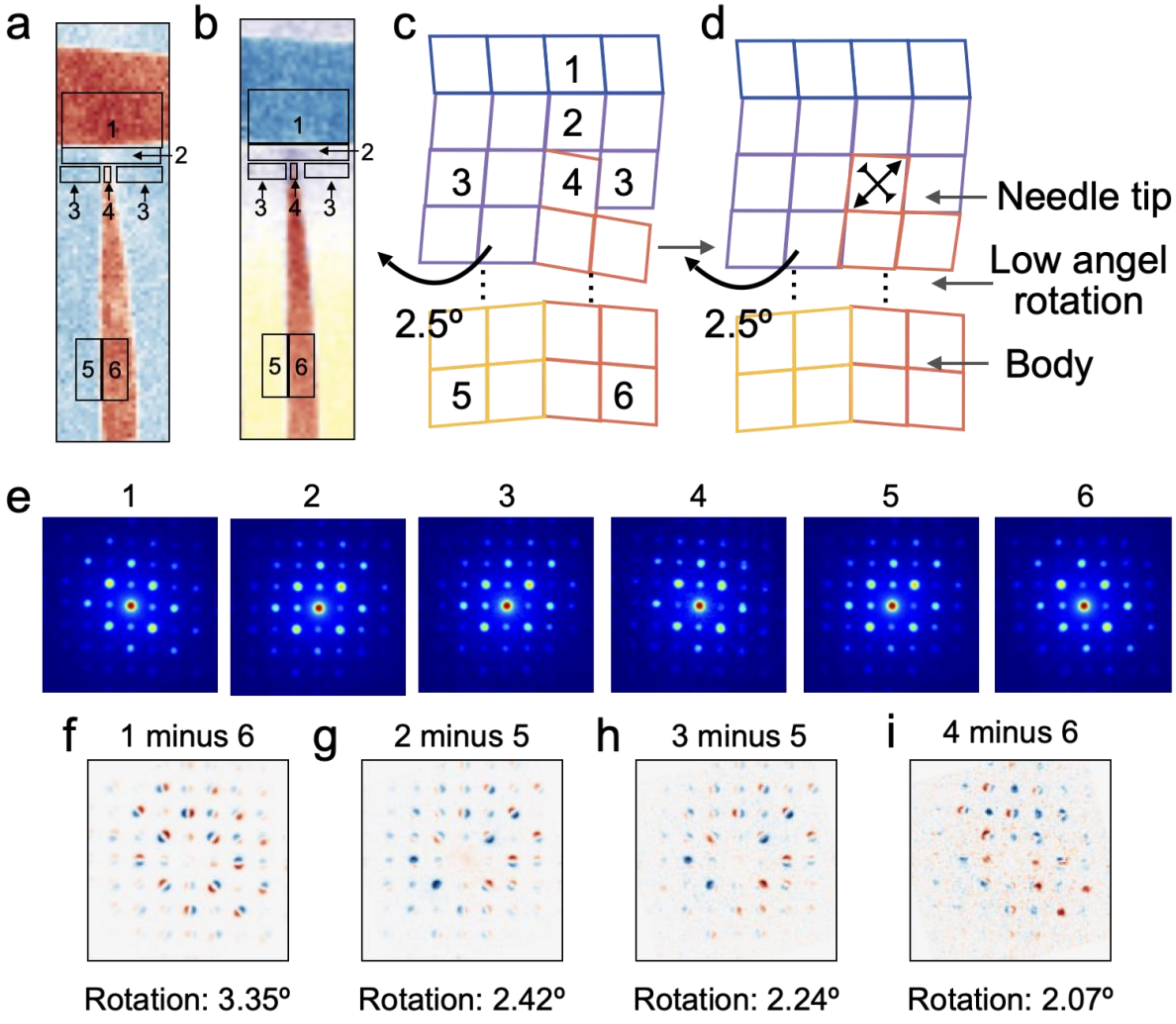

Figure S15 | Strain and rotation at needle tips. (a,b), Ferroelastic strain and rotation maps of a needle tip structure. **(c,d)**, Lattice schematic of the needle tip with rigid model (c) and deformed model (d). **(e)** Diffraction patterns from the six boxed regions in (a and b). **(f - i)** Diffraction differences with the relative rotation angle labeled.

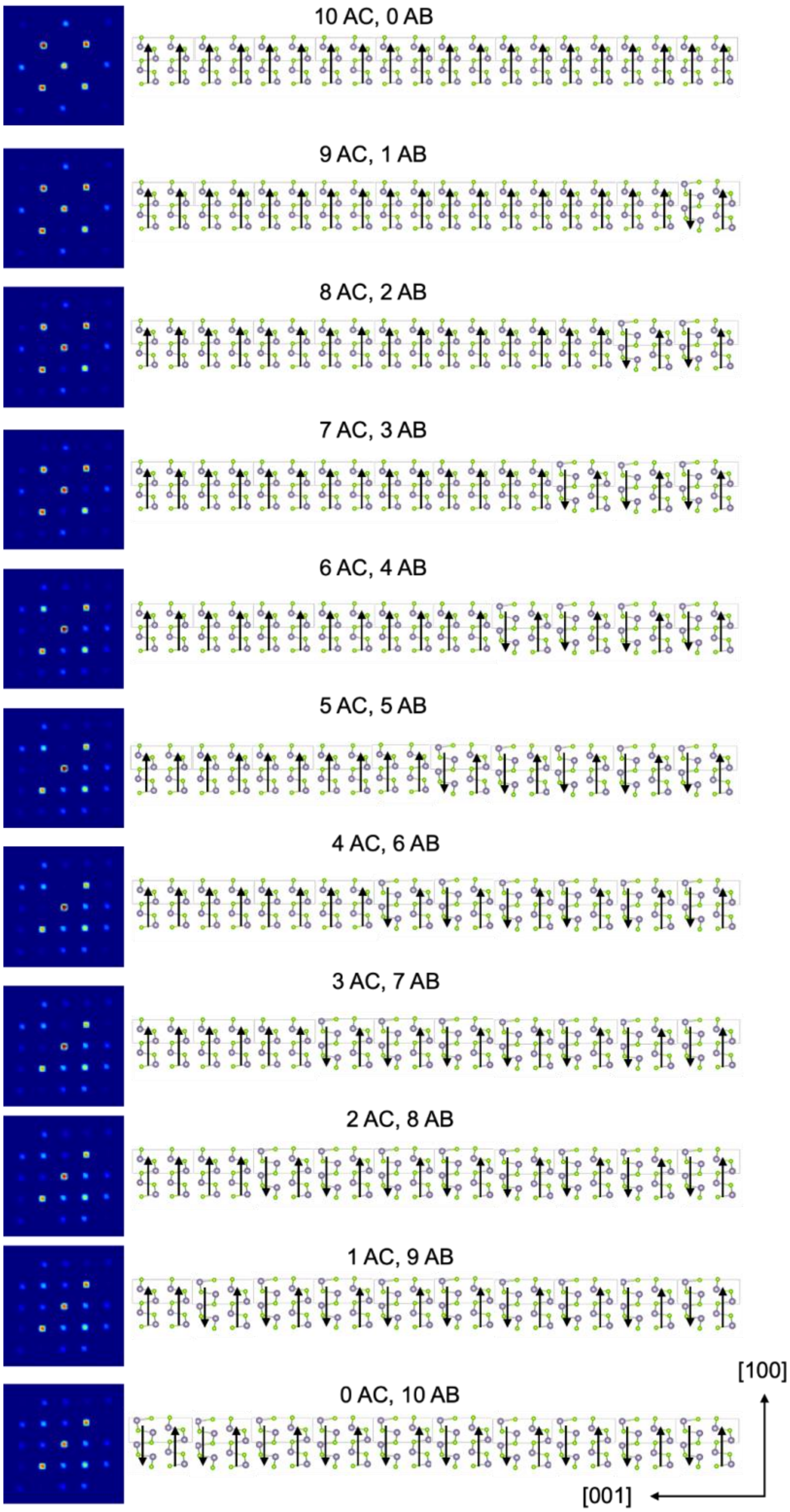

Figure S16 | Multislice simulation of diffraction patterns with different stacking sequence.

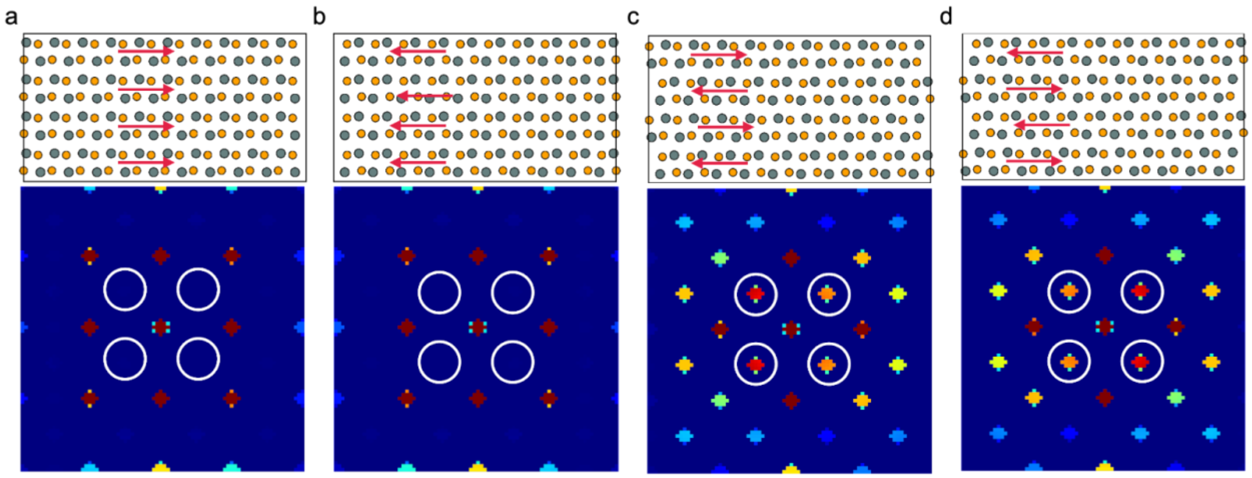

Figure S17 | Multislice simulation of diffraction patterns from pure AC and AB stacking. (a-b) AC stacking with the opposite polarization directions. **(c-d)** AB stacking with sequences "A-B" and "B-A". In the "A-B" sequence (c), the {110} spots on the left are brighter than those on the right, while the opposite is true in the "B-A" sequence (d). To reduce the errors from this unbalanced intensity, we sum the intensities of the four {110} spots.

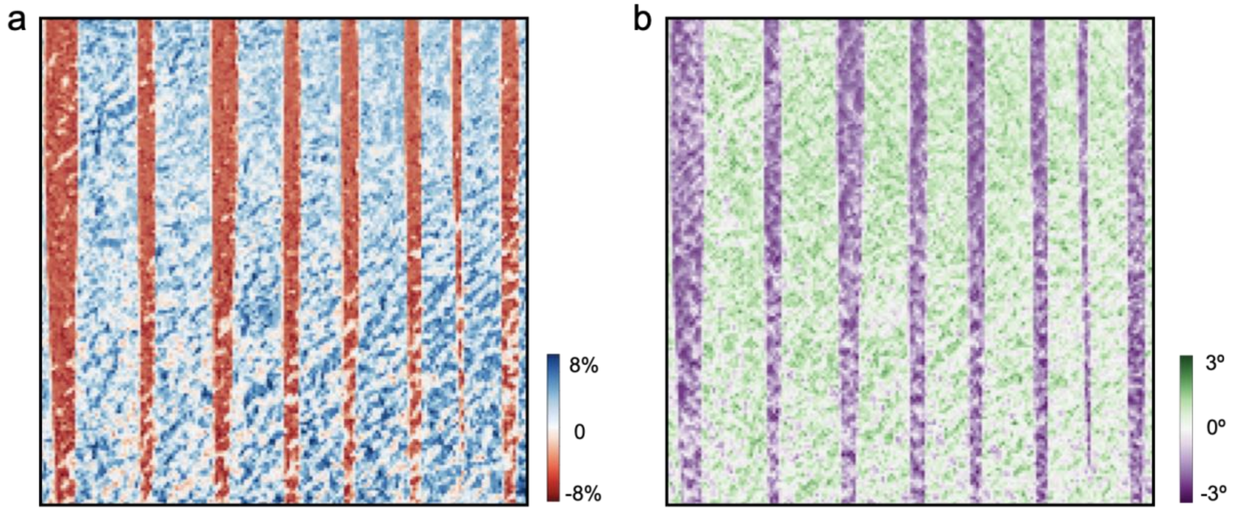

Figure S18 | Strain map (a) and rotation map (b) from the sample area shown in Figure 4(c).

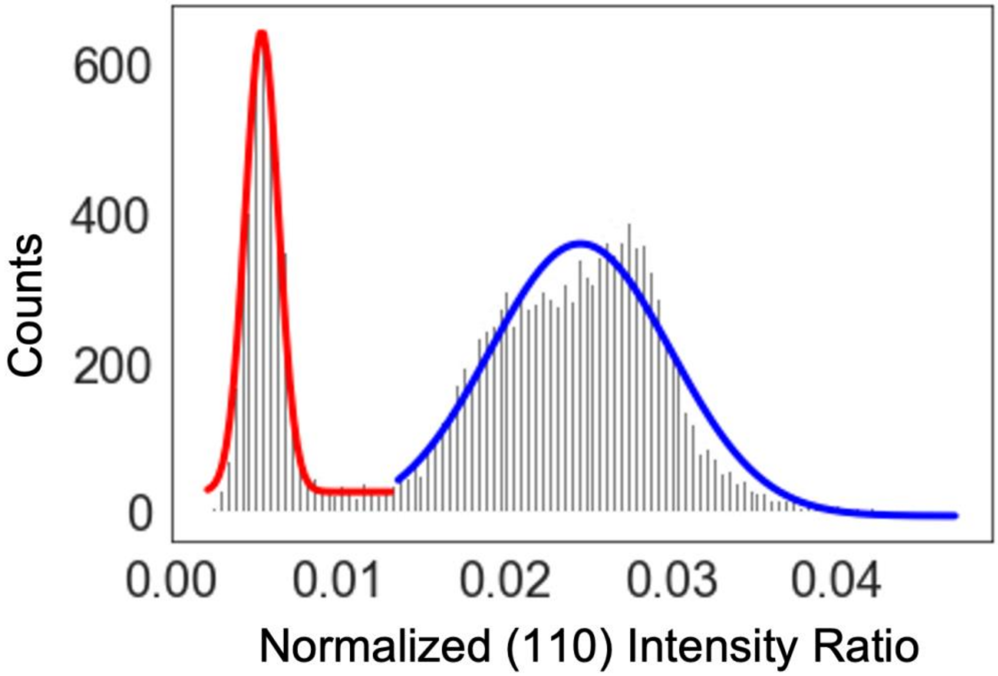

Figure S19 | Histogram of the normalized intensity map from Figure 4c. The red peak is at 0.0054 and the blue peak is at 0.025.

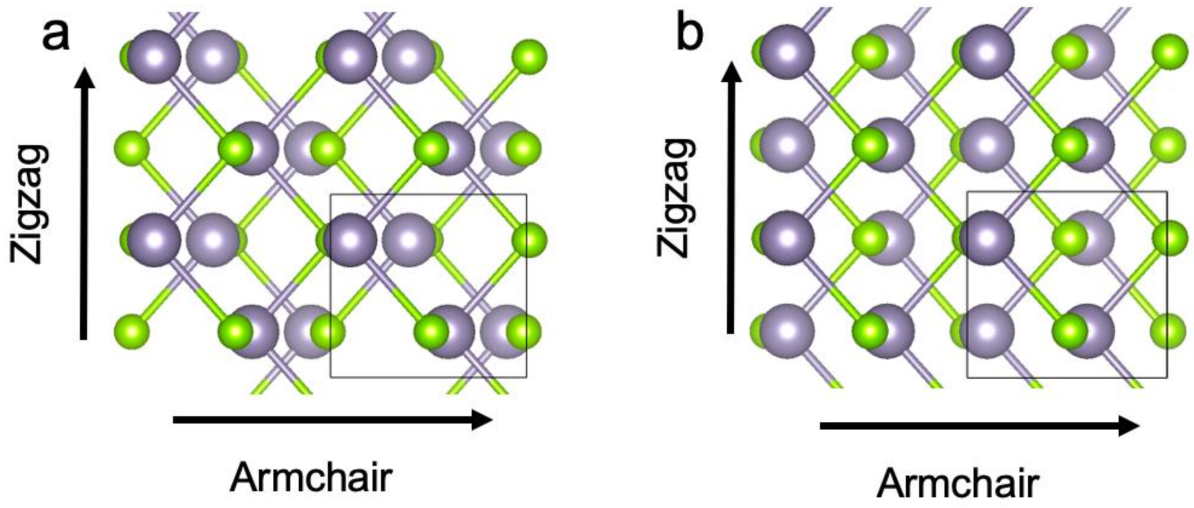

Figure S20 | Lattice schematic of AFE and FE stacking SnSe. (a) In-plane lattice schematic of the AFE phase, which has a “dumbbell” shape. **(b)** In-plane lattice schematic of the FE phase, which has a “square” shape.

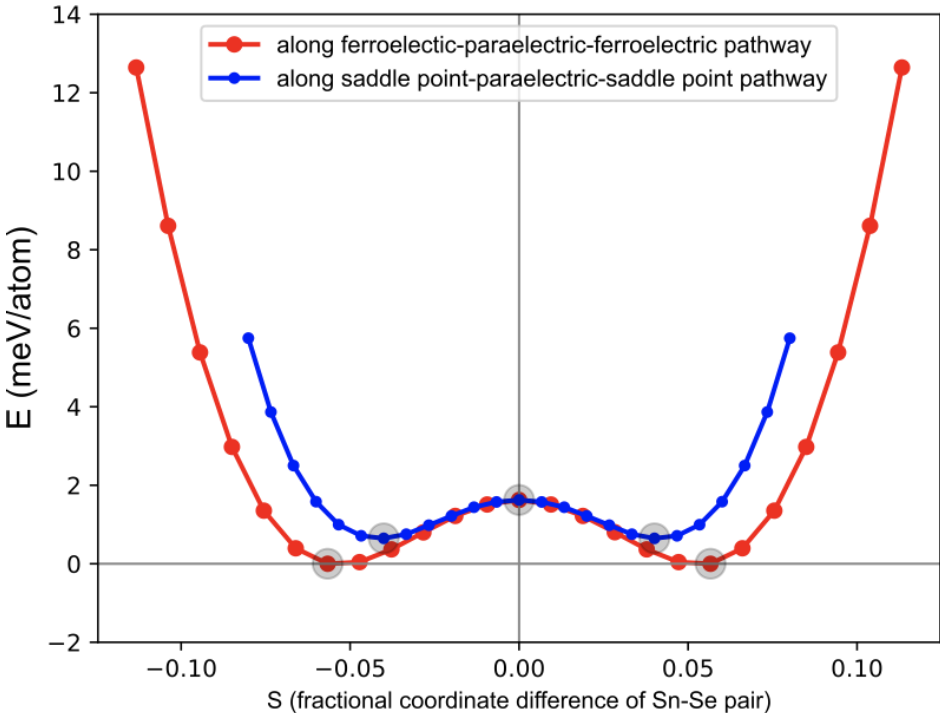

Figure S21 | Transition energy pathway of monolayer SnSe as a function of the fractional coordinate difference of the Sn-Se pair under two distinct transformation paths. Red curve shows the minimum energy pathway through ferroelectric-paraelectric-ferroelectric structures, where two global minima (ground-state ferroelectric phase) are indicated by the shaded gray dots. Blue curve shows another energy pathway along the saddle point-paraelectric-saddle point structures, where two saddle points are indicated by another two shaded gray dots. Paraelectric phase is indicated by a shaded dot at S=0. The data clearly shows that the ferroelectric transformation prefers to take place via Sn-Se pair rotation without going through the energetically unstable paraelectric phase.

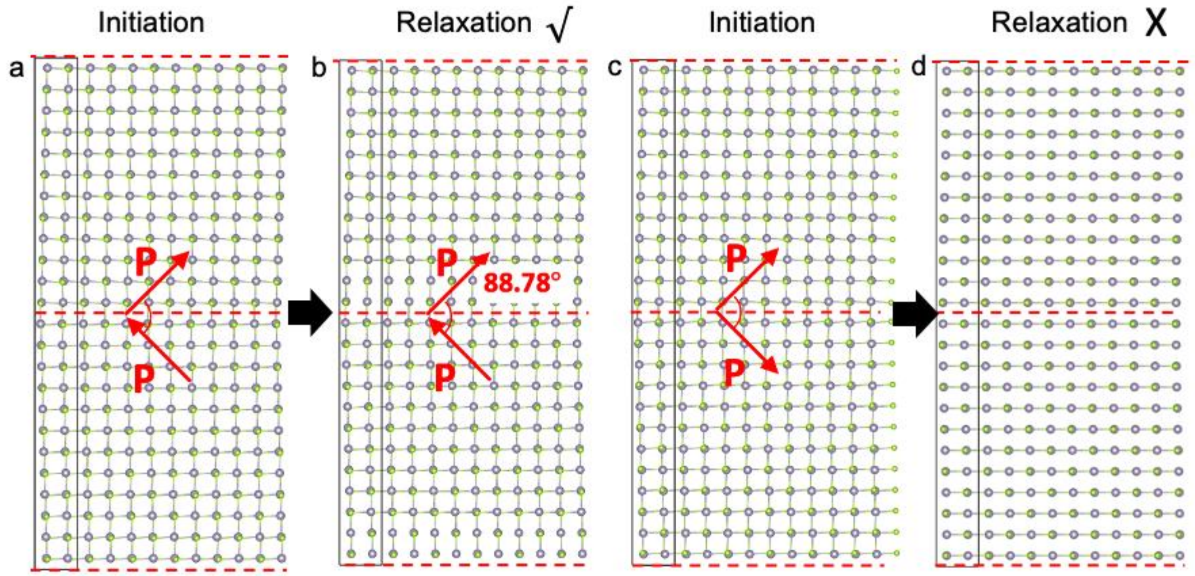

Figure S22 | DFT simulation of twin walls in a monolayer SnSe. (a) Initial atomistic structure of head-to-tail twin boundary. **(b)** Relaxed atomistic structure of head-to-tail twin boundary. **(c)** Initial atomistic structure of tail-to-tail twin boundary (or head-to-head due to the periodic boundary condition). **(d)** Relaxed atomistic structure of tail-to-tail twin boundary.

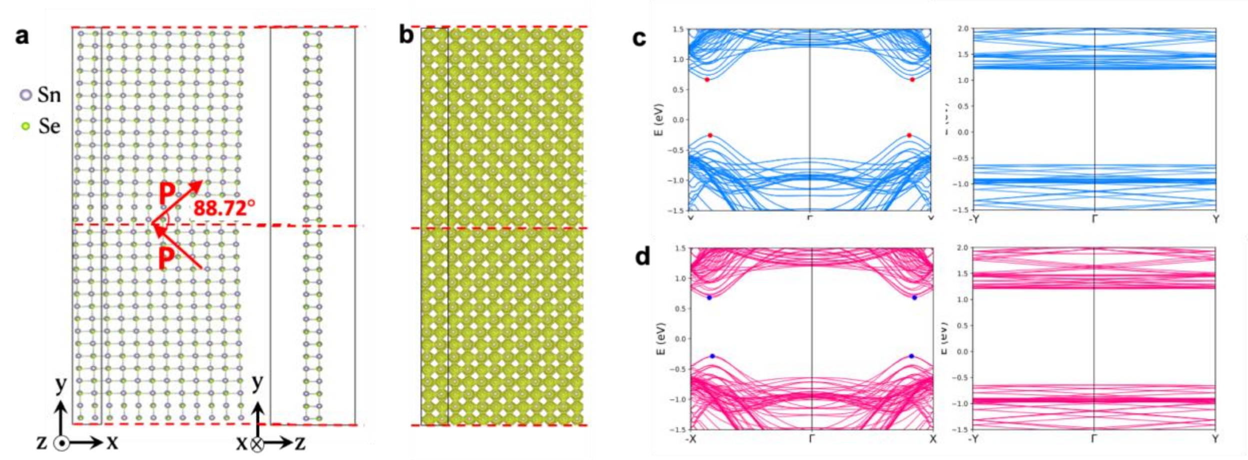

Figure S23 | DFT simulation of twin walls in a monolayer SnSe. (a) Relaxed atomistic structure of head-to-tail twin boundary with angle of 88.72°. **(b)** Isosurface of charge density for the corresponding monolayer SnSe with head-to-tail twin boundary. **(c,d)** Electronic band structure of monolayer SnSe with and without the twin boundary, respectively.

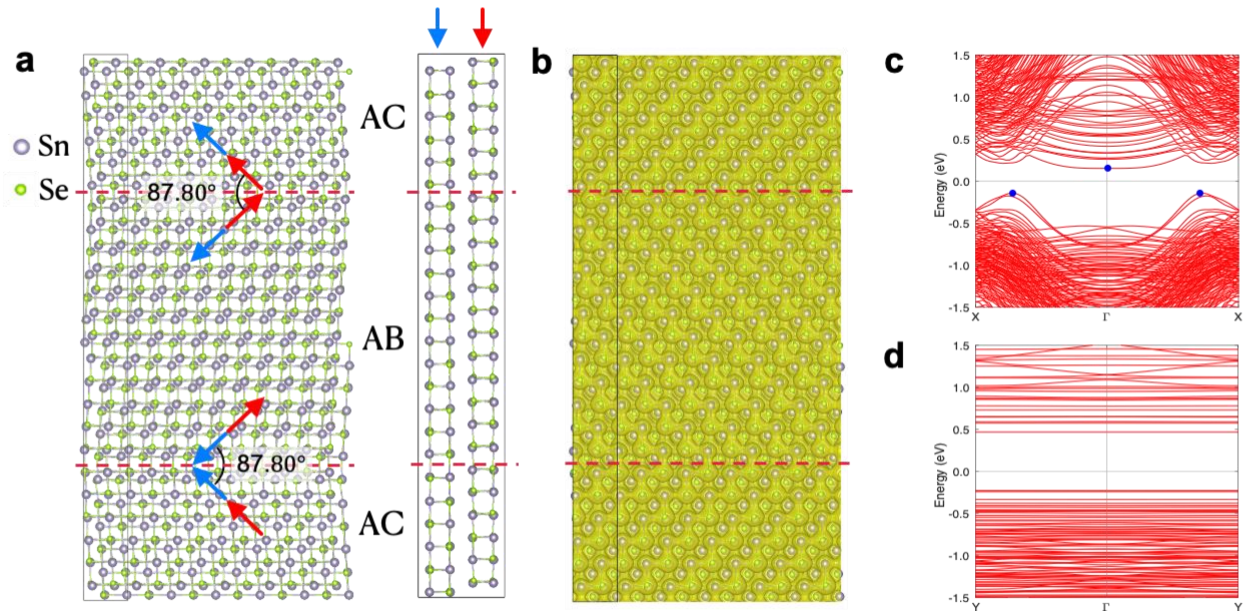

Figure S24 | DFT simulation of AFE-FE twin boundary. (a) Relaxed atomistic structure of AFE-FE (AB-AC) twin boundary with an angle of 87.80°. The twin boundary is indicated by a red dashed line. Red and blue arrows indicate the electric polarization direction in the x-y plane for top and bottom layer, respectively. **(b)** Isosurface of charge density for the corresponding bulk SnSe with AB-AC twin boundary. **(c,d)**, Electronic band structure of bulk SnSe with the AB-AC twin boundary.

	4D-STEM		FE-AFE boundary from DFT		Bilayer lattice from ref[2]		Monolayer[3]
Stacking	AC	AB	AC	AB	AC	AB	
Armchair (Å)	4.42	4.39	4.46	4.45	4.370	4.365	4.35
Zigzag (Å)	4.23	4.28	4.28	4.30	4.210	4.219	4.26
Ferroelastic strain	4.6%	2.6%	4.2%	3.4%	3.80%	3.46%	2.1%

Table S1 | Lattice constant and ferroelastic strain of SnSe from experiment and simulation. The red numbers indicate that the structure of the DFT calculated FE-AFE domain wall closely resembles the experimental results obtained from our 4D-STEM.

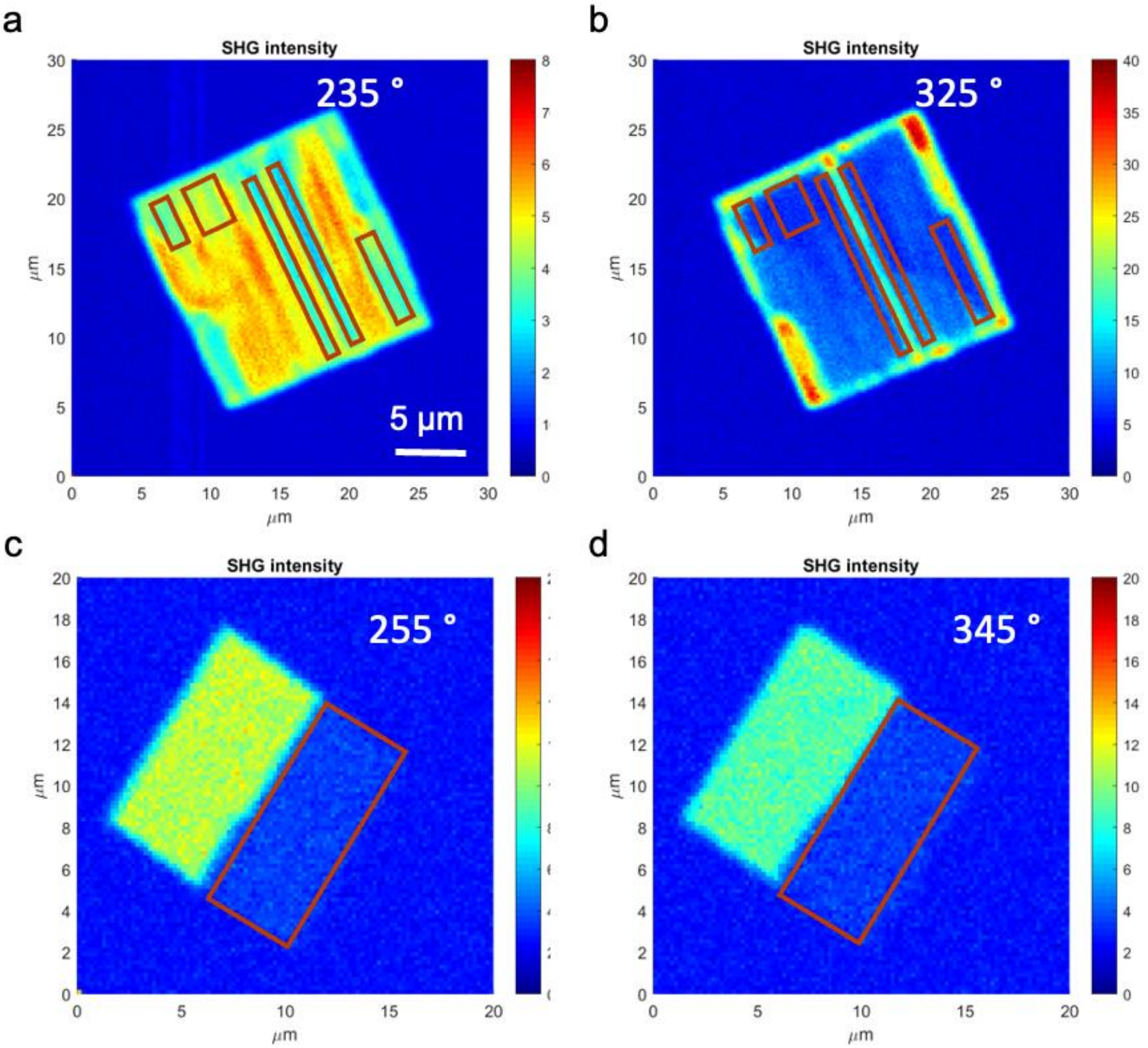

Figure S25 | SHG images of as-grown SnSe thin flakes. (a-b) SHG intensity maps, taken at different angles, from an as-grown SnSe on a mica substrate, showing areas with reduced intensity (red boxes). These areas indicate predominant AB stacking (AFE phase). **(c-d)** SHG intensity maps from a different as-grown SnSe flake on mica. The regions (red box) with diminished SHG intensity indicate areas of AB stacking (AFE phase). In contrast, areas with high SHG intensity regions correspond to AC stacking (FE phase). The observations confirm that as-grown SnSe inherently exhibits a coexistence of both AB and AC stacking.

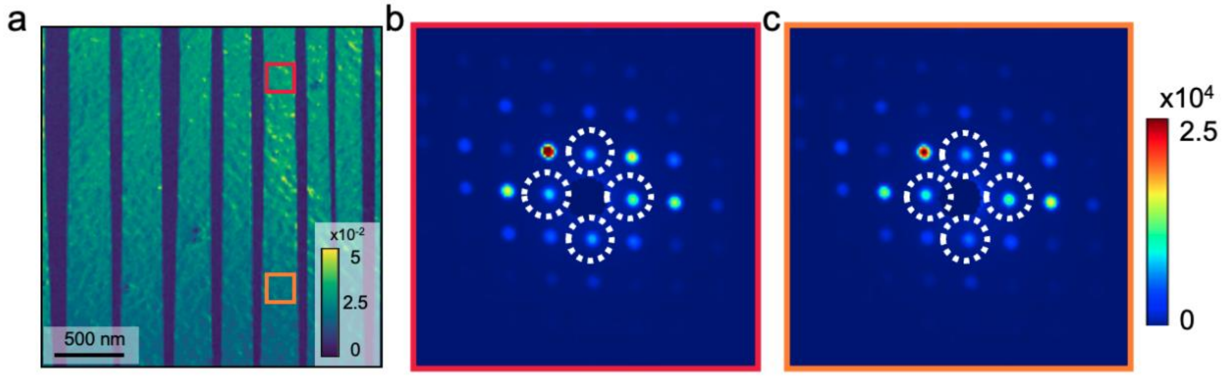

Figure S26 | Lattice tilt effect in the SnSe flake. (a) Normalized intensity ratio map from **Figure 4c**. **(b-c)** Mean diffraction patterns from the red and orange boxes in (a), respectively. The {110} intensity is uneven in the AB stacking region due to a small lattice tilt in the SnSe flake. The summed intensity from the circled spots in panel (b) is slightly brighter than in panel (c), causing the intensity variation observed in panel (a), and contributing to the broad blue peak in **Figure S19**. In contrast, in AC stacking domains, the forbidden {110} spots are nearly absent, so the lattice tilt does not affect the intensity ratio. This results in the narrow red peak in **Figure S19**.

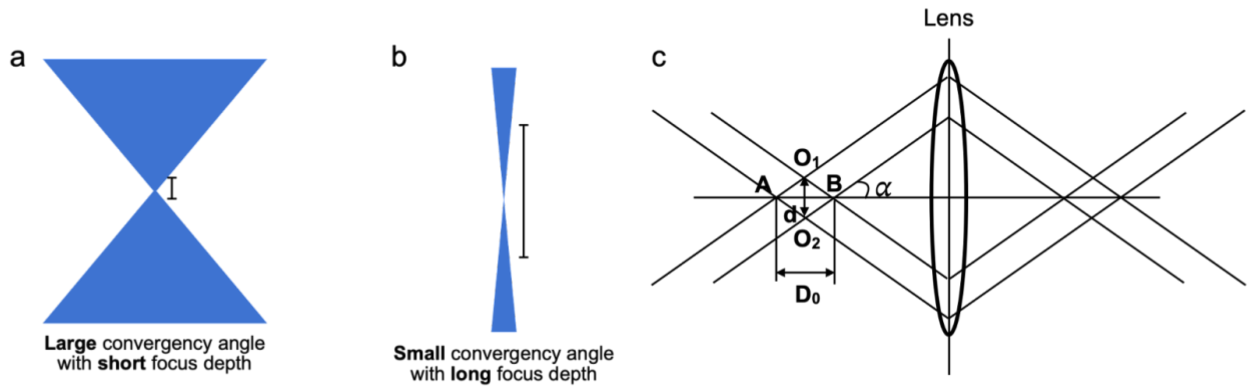

Figure S27 | EMPAD data acquisition conditions. (a) Conventional STEM with a large convergence angle, leading to a smaller probe and a reduced depth of focus. **(b)** Nanobeam STEM with a small convergence angle, resulting in a larger probe and an increased depth of field. **(c)** Schematic illustrating the probe size and depth of field. The probe size, d, is given by $0.61\lambda/\alpha$, where λ represents the wavelength of the electron beam, and α is the convergence angle. The depth of focus, D_0, is calculated by $d/\tan\alpha \approx 0.61\lambda/\alpha^2$. For our experimental conditions, we utilized a 0.5 mrad convergence angle and a 300 keV electron beam. As a result, the probe size is 2.44 nm (defined by Full Width Half Maximum (FWHM) probe diameter), and the depth of field is 4.88 µm.

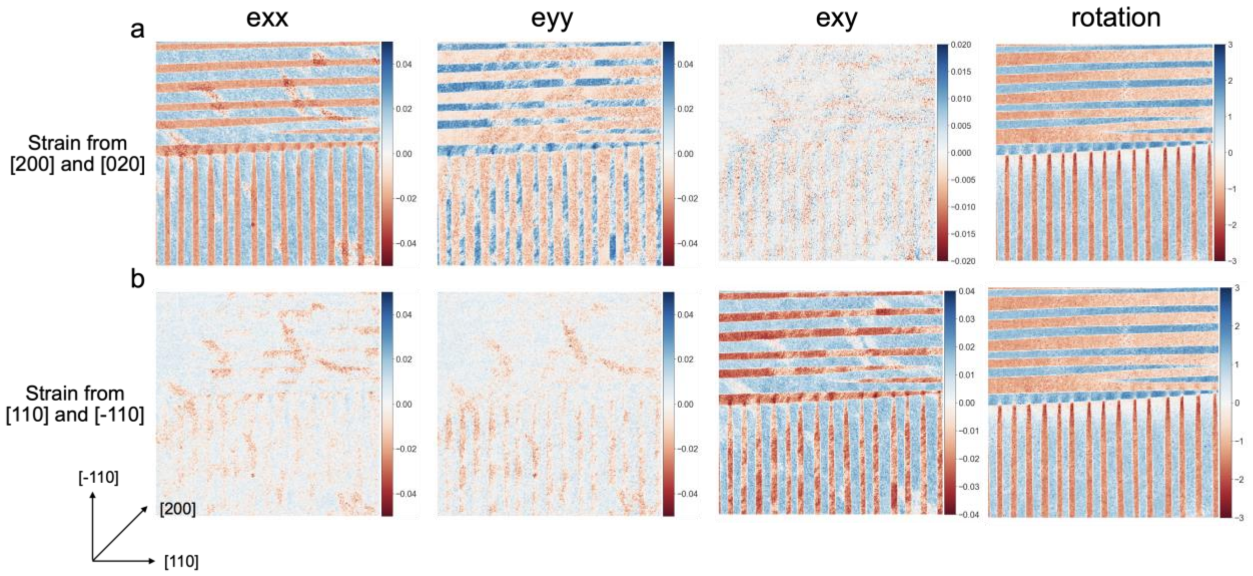

Figure S28 | Strain maps from the polar decomposition approach. **(a)** Strain maps using [200] and [020] as the basis vectors. The e_{xx} and e_{yy} maps show the uniaxial strain along [200] and [020]. The e_{xy} map shows minimal contrast, indicating the [200] and [020] vectors remain mostly orthogonal. The rotation map illustrates the lattice rotation of the two vectors. **(b)** Strain maps utilizing [110] and [-110] as the basis vectors. The e_{xx} and e_{yy} maps exhibit minimal contrast, indicating minor strain along [110] and [-110]. The e_{xy} map shows the diagonal deformation, which is along [200] and [020]. Therefore, the e_{xy} map shows the similar contrast with the ferroelastic map, the detailed explain in **Figure S29**. The rotation map remains consistent with the one in (a).

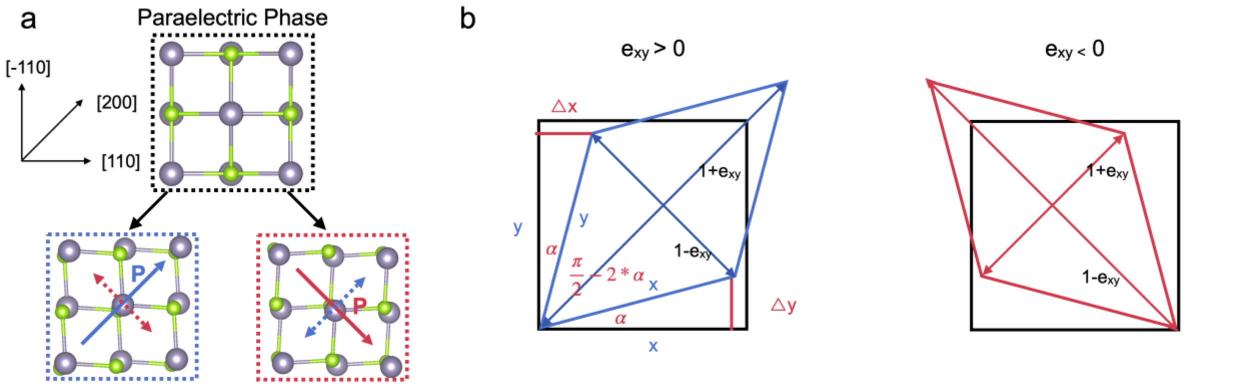

Figure S29 | Relationship between shear strain and ferroelastic strain. (a) Schematic of the SnSe lattice during phase transition. SnSe lattices can elongate in either the [200] direction or the [020] direction. **(b)** Schematic illustrating the relationship between shear strain and ferroelastic strain. Due to lattice elongation along the diagonal ([200]) direction, the unstrained (or squared) shape lattice transforms into a rhombus-shaped lattice. This transformation induces a small shear angle, α, as labeled in (b). Given that the uniaxial strain along x ([110]) and y ([-110]) directions is closed to zero (**Fig. S28b**) and $\boldsymbol{\alpha}$ is small:

$$\alpha \approx sin\alpha = \frac{\Delta x}{y} = \frac{\Delta y}{x} = \frac{1}{2}\left(\frac{\Delta x}{y} + \frac{\Delta y}{x}\right) = e_{xy}\,, \qquad [1]$$

where e_{xy} is the shear strain. Meanwhile, the [200] lattice constant can be expressed as:

$$d_{200} = x\ \times cos\left(\frac{\pi}{4} - \alpha\right) \times 2 = x \times \left(\frac{\sqrt{2}}{2} \times cos\alpha + \frac{\sqrt{2}}{2} sin\alpha\right) \times 2 = \sqrt{2}\,x(cos\alpha + sin\alpha) \; [2]$$

Since $\boldsymbol{\alpha}$ is small, $cos\alpha \approx 1, sin\alpha = \alpha$. Therefore, the diagonal lattice constants are:

$$d_{200} = \sqrt{2}\,x(1+\alpha) = \sqrt{2}\,x(1+e_{xy}) \qquad [3]$$

$$d_{020} = \sqrt{2}\,x(1-\alpha) = \sqrt{2}\,x(1-e_{xy}) \qquad [4]$$

And the ferroelastic strain is:

$$\frac{d_{200}}{d_{020}} - 1 = \frac{1+e_{xy}}{1-e_{xy}} - 1 = \frac{2e_{xy}}{1-e_{xy}} \approx 2e_{xy} \qquad [5]$$

In terms of lattice rotation, we utilized the "armchair rotation", which refers to the rotation angle of the elongated diagonal lattice direction. As a result, the two cases presented in (b) exhibit a ~90º rotation. In contrast, the conventional polar decomposition strain mapping method (**Fig. S28**) cannot extract this information.

References

1. Han, Y. *et al.* Strain Mapping of Two-Dimensional Heterostructures with Subpicometer Precision. *Nano Lett* **18**, 3746–3751 (2018).

2. Xu, B., Deng, J., Ding, X., Sun, J. & Liu, J. Z. Van der Waals force-induced intralayer ferroelectric-to-antiferroelectric transition via interlayer sliding in bilayer group-IV monochalcogenides. *Npj Comput Mater* **8**, 47 (2022).

3. Chang, K. *et al.* Microscopic Manipulation of Ferroelectric Domains in SnSe Monolayers at Room Temperature. *Nano Lett* **20**, 6590–6597 (2020).
